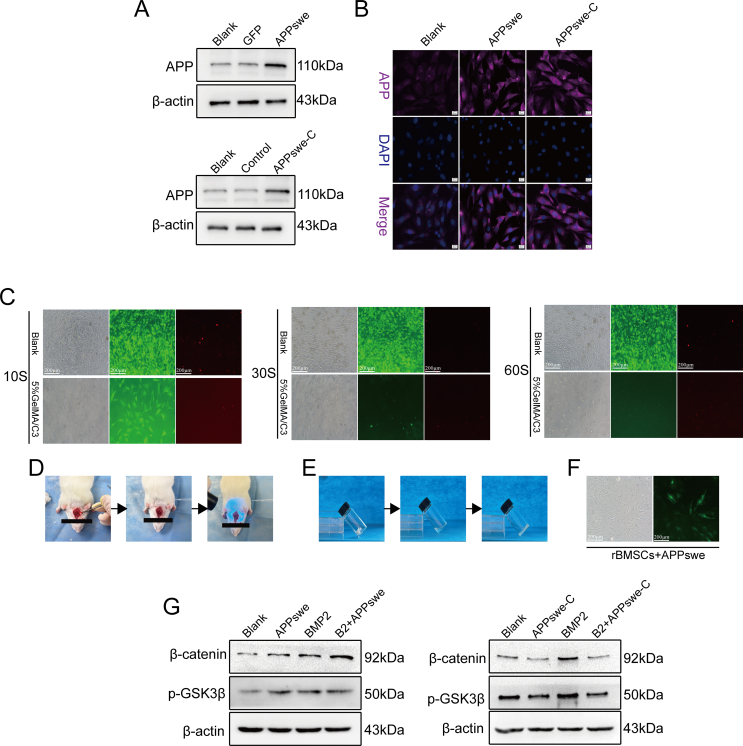# The bidirectional effects of APPswe on the osteogenic differentiation of MSCs in bone homeostasis by regulating Notch signaling

**DOI:** 10.1016/j.gendis.2024.101317

**Published:** 2024-05-09

**Authors:** Nan Wang, Xiaoyu Shen, Huakun Huang, Runhan Zhao, Habu Jiwa, Zongxin Li, Pei Li, Jixing Ye, Qiang Zhou

**Affiliations:** a Department of Orthopedics, The Third Affiliated Hospital of Chongqing Medical University, Chongqing 401120, China; b Key Laboratory of Clinical Laboratory Diagnostics, Ministry of Education, Chongqing Medical University, Chongqing 400016, China; c Chengdu University, Chengdu 610106, China; d Department of Orthopedics, The First Affiliated Hospital of Chongqing Medical University, Chongqing 400042, China

**Keywords:** Alzheimer's disease, Amyloid precursor protein, MSCs, Notch signaling, Osteogenic differentiation

## Abstract

Amyloid precursor protein (APP), especially Swedish mutant APP (APPswe), is recognized as a significant pathogenic protein in Alzheimer's disease, but limited research has been conducted on the correlation between APPswe and the osteogenic differentiation of mesenchymal stem cells (MSCs). The effects of APPswe and its intracellular and extracellular segments on the osteogenic differentiation of bone morphogenetic protein 2 (BMP2)-induced MSCs were analyzed in this study. Our analysis of an existing database revealed that APP was positively correlated with the osteogenic differentiation of MSCs but negatively correlated with their proliferation and migration. Furthermore, APPswe promoted BMP2-induced osteogenic differentiation of MSCs, while APPswe-C (APPswe without an intracellular segment) had the opposite effect; thus, the intracellular domain of APPswe may be a key factor in promoting the osteogenic differentiation of MSCs. Additionally, both APPswe and APPswe-C inhibited the proliferation and migration of MSCs. Furthermore, the intracellular domain of APPswe inhibited the activity of the Notch pathway by regulating the expression of the Notch intracellular domain to promote the osteogenic differentiation of MSCs. Finally, APPswe-treated primary rat bone marrow MSCs exhibited the most favorable bone repair effect when a GelMA hydrogel loaded with BMP2 was used for *in vivo* experiments, while APPswe-C had the opposite effect. These findings demonstrate that APPswe promotes the osteogenic differentiation of MSCs by regulating the Notch pathway, but its extracellular segment blocks the self-renewal, proliferation, and migration of MSCs, ultimately leading to a gradual decrease in the storage capacity of MSCs and affecting long-term bone formation.

## Introduction

With the increasing global average life expectancy and the growing prevalence of aging, age-related degenerative diseases will place a significant burden on public health resources. Alzheimer's disease (AD), a major age-related neurodegenerative disease, currently affects 10% of adults aged 65 and older, and it is estimated that the global incidence rate will reach 132 million cases in the next 30 years.^1^ Existing research has focused mainly on the damage caused by AD to the nervous system while neglecting the impact of AD on other systems. In recent years, statistical data from various regions and populations have shown that AD patients have a risk of decreased bone density and fractures that is more than twice as high as that of age-matched individuals in normal populations.^2^^,^^3^ Fractures significantly impact the quality of life of AD patients, increase their postoperative mortality rate, and impose a medical burden.^2^ The traditional view is that this situation may be related to factors such as reduced activity, insufficient sunlight exposure, and an increased tendency to fall in AD patients.^4^^,^^5^ AD is characterized by long latency and slow progression,^6^ and its neuropathological changes occur 30 years before clinical manifestation.^7^ Bone loss in patients occurs 8–10 years before the onset of cognitive impairment and its diagnosis, indicating that abnormal expression of Swedish mutant APP (APPswe) and amyloid-beta peptide (Aβ) deposition may also be associated with bone loss.^8^ Cognitive impairment and mobility limitations in AD patients often occur in the later stages of the disease, as supported by research.^9^, ^10^, ^11^ Even after controlling for factors associated with falling tendencies, the fracture rate of AD patients is still much greater than that of normal people, which indicates that bone loss and osteoporosis are the fundamental causes of fracture, rather than cognitive impairment or falling tendencies.^12^^,^^13^ At the same time, an increasing number of viewpoints suggest that the pathological changes in AD are closely related to bone mass loss,^2^^,^^14^ and the two diseases may have common pathogenic mechanisms.

Amyloid precursor protein (APP) is expressed in a variety of cells, including bone marrow mesenchymal stem cells (BMSCs), osteocytes, and osteoclasts. APP undergoes multiple cleavages by α-, β-, and γ-secretases, resulting in the generation of extracellular fragments known as Aβ and the intracellular domain of APPswe (AICD).^15^ Most of the existing research has focused on the role of APP in the nervous system, whereas there have been few controversial studies on its role in the skeletal system. Some studies have indicated that APP positively regulates osteogenic differentiation, while other studies have indicated that APPswe inhibits the osteogenic differentiation of mesenchymal stem cells (MSCs) by regulating oxidative stress.^16^^,^^17^ Similarly, it is believed that Aβ promotes osteogenic differentiation and inhibits osteoclast activity by regulating certain pathways,^14^ while some studies have shown that Aβ inhibits the osteogenic differentiation and proliferation of MSCs and promotes osteoclast activity.^2^^,^^8^^,^^18^, ^19^, ^20^, ^21^

AICD is susceptible to degradation by various enzymes present in the cytoplasm.^22^^,^^23^ AICD can exert regulatory effects by binding to a variety of transcription factors,^23^ and these interactions have been found to play roles in the regulation of cell proliferation and apoptosis.^22^, ^23^, ^24^, ^25^ However, there have been few studies on the relationship between AICD and bone formation. Many studies have suggested that AICD and NICD (Notch intracellular domain) may have similar transcriptional functions,^26^ for example, AICD can bind with NICD and RBP-jk (a recombination signal binding protein for the immunoglobulin kappa J region), accelerate their degradation, and subsequently inhibit the activity of the Notch pathway.^27^, ^28^, ^29^ The general view is that the Notch pathway inhibits the osteogenic differentiation of MSCs, promotes their proliferation, and maintains their storage and stem cell characteristics.^26^^,^^30^ When the Notch1/2 gene was knocked out, the osteogenic differentiation of MSCs was enhanced, and the bone mass increased in the early stage, while the bone mass significantly decreased over time, which was related to the retardation of MSC proliferation and the gradual decrease in MSC storage.^31^ Interestingly, studies have also shown that the expression levels of the osteoblastic differentiation-related protein osteopontin (OPN) in the cerebrospinal fluid and serum of AD patients are abnormally elevated,^32^^,^^33^ changes that occur much earlier than the onset of AD dementia symptoms, and patients show simultaneous decreases in bone density and bone mass.^13^^,^^34^ According to the above results, it is hypothesized that the Notch signaling pathway may be closely related to bone mineral density reduction and osteoporosis in AD patients. Moreover, it is generally believed that the balance between an individual's osteogenic differentiation ability and osteoclast activity mainly determines bone homeostasis, but the importance of stem cell characteristics such as the self-renewal, migration, and proliferation abilities of MSCs is unknown.^31^^,^^35^ When the above-mentioned abilities are affected, low bone density and osteoporosis can occur. Therefore, we speculated that AICD may inhibit the Notch pathway by binding to NICD, thereby promoting the osteogenic differentiation of MSCs in the early stage of AD but inhibiting their self-renewal and proliferation, resulting in the gradual depletion of MSCs' storage and long-term bone formation disorders.

In this study, using the Gene Expression Omnibus (GEO) database, we determined that APP was positively correlated with the osteogenic differentiation of MSCs but negatively correlated with the proliferation and migration of MSCs. Furthermore, APPswe promoted the osteogenic differentiation of MSCs induced by bone morphogenetic protein 2 (BMP2, sometimes referred to as B2), and APPswe or APPswe-C (APPswe without an intracellular segment) was negatively correlated with MSCs proliferation and migration *in vitro*. In addition, we found that APPswe affects the osteogenic differentiation and proliferation of MSCs by regulating the expression and nuclear translocation of NICD. Through the above studies, we hope to clarify the role and mechanism of APPswe in the bone formation of MSCs and provide a theoretical basis for the early diagnosis and treatment of bone loss in AD patients, as well as the enhancement of fracture repair capability in AD patients.

## Materials and methods

### Cell culture and chemicals

C3H10T1/2 cells were derived from laboratory preservation, which were obtained from ATCC (American Type Culture Collection, Manassas, VA) and were verified by STR analysis. C3H10T1/2 cells were cultured at 37 °C in complete Dulbecco's modified Eagle's medium supplemented with 10% fetal bovine serum, 1% penicillin, and 1% streptomycin in a 5% CO_2_ incubator under a humidified atmosphere. Recombinant adenovirus expressing exogenous human APP695 with double mutations at KM670/671NL (Swedish mutations, called Ad-APPswe) was purchased from Hanheng Biological Technology (Shanghai, China) and the APPswe overexpression plasmid without the intra-membrane fragment (APP1-638, called APPswe-C) was designed and synthesized by Leqin Biotechnology (Chongqing, China), and the expression of APPswe and APPswe-C in MSCs were verified by western blot and cellular immunofluorescence assays (Figs. S1A and B). Recombinant adenovirus expressing exogenous NICD (Ad-NICD) is derived from laboratory preservation. Recombinant protein BMP2 was purchased from Sino Biological (Beijing, China). Dulbecco's modified Eagle's medium was purchased from Saimike Biotechnology (Chongqing, China), and fetal bovine serum was obtained from Cellmax (Beijing, China). anti-OCN (osteocalcin), anti-MMP9 (matrix metallopeptidase 9), anti-MMP7 (matrix metallopeptidase 7), anti-E-cadherin, anti-N-cadherin, anti-Snail, anti-p-GSK3β (Ser9), anti-p-Smad1/5/8, and anti-cyclin D1 antibodies were purchased from Affinity Biosciences (Changzhou, China). anti-Runx2, anti-β-catenin, anti-Lamin B1, anti-c-Myc, anti-OPN, and anti-PCNA antibodies were obtained from Bimake Biotechnology (Houston, USA) and anti-β-actin antibody was purchased from Proteintech (Wuhan Sanying Biological Technology, Wuhan, China). Anti-NICD antibody was purchased from ImmunoWay Biotechnology (Suzhou, China). FITC anti-rat CD45, PE anti-rat CD90, and PE anti-rat CD29 antibodies were obtained from Elabscience Biotechnology (Wuhan, China). GelMA's photoinitiator Lithium Pheny l (2,4,6-trimethylbenzoy l) phosphinate was obtained from Bidepharm (Bide Medical Technology, Shanghai, China). Gelatin methacryloyl (GelMA) was obtained from CureGel (Shuhe Biotechnology, Wenzhou, China).

### Gene analysis based on the GEO database

Gene expression data for human BMSCs during the mineralization process were downloaded from the GEO database (https://www.ncbi.nlm.nih.gov/geo/), aiming to explore the molecular mechanism of APP in osteogenic transformation. First, the changes in the expression of APP, the osteogenic marker gene alkaline phosphatase (ALP), and the osteoclast marker gene acid phosphatase 5 (ACP5) were explored during osteogenic transformation. Moreover, the correlations between APP and ALP and between APP and ACP5 were explored via Pearson correlation analysis. Finally, the correlations between APP and the proliferation-related genes PCNA (proliferating cell nuclear antigen) and CCND1 (cyclin D1) and the migration-related genes MMP7 and MMP9 were explored via Pearson correlation analysis.

### Cell transfection

Cells were plated in 100 mm petri dishes or well plates. When the cells reached 60%–70% confluence, they were transduced with the APPswe-C plasmid, Ad-APPswe, or Ad-NICD using Lipofectamine TM 3000 (Invitrogen, Gaithersburg, MD) or Polybrene® (Santa Cruz Biotechnology, Shanghai, China), respectively, according to the manufacturers' recommendations.

### ALP assays

The ALP activity of the cells was assessed with an alkaline phosphatase activity assay kit (Beyotime, Shanghai, China) according to the manufacturer's instructions, and the absorbance was measured at 405 nm using a microplate reader. ALP staining was performed using the BCIP/NBT alkaline phosphatase color development kit (Beyotime, Shanghai, China) according to the manufacturer's recommendations. Each assay condition was performed in triplicate, and the results were repeated in at least three independent experiments.

### Alizarin red S (ARS) staining

To assess mineralized nodule formation, cells were seeded in 24-well plates and cultured in the presence of ascorbic acid (50 mg/mL) and glycerophosphate (10 mM) after being transduced with the APPswe-C plasmid, Ad-APPswe, or Ad-NICD. Then, the cells were treated with BMP2 after transfection for 24 hours (h). The culture media were changed one day apart, BMP2 was added, and bone nodule formation was assessed by calcium precipitation through staining with ARS solution (Solarbio, Beijing, China) after culturing for 21 days. After several washes with phosphate buffer saline solution (PBS), the stained nodules were desorbed with 200 μL of 10% cetylpyridinium chloride (Aladdin Biochemical Technology, Shanghai, China) for 20 minutes (min), and the absorbance was detected at 590 nm.

### Immunofluorescence staining

The cells were cultured in 60 mm dishes to approximately 60% confluence and then transfected with APPswe or APPswe-C. The cells were then collected and counted after 24 h. The 14 mm crawling tablets were placed in a 24-well plate, and the crawling tablets were moistened with 0.1% gelatine and then placed in an incubator at 37 °C for 30 min. Then, 50,000 treated cells were spread into the wells containing the crawling tablets, and BMP2 was added to the corresponding group. After 36 h of culture, the medium was discarded, and the coverslips were washed with PBS three times for 3–5 min each. The cells were fixed with 4% paraformaldehyde for 30 min and washed with PBS. Then, the cells were treated with 0.5% Triton X-100 (Solarbio, Beijing, China) and blocked with 1% bovine serum albumin (Solarbio, Beijing, China). After washing with PBS, the cells were incubated with primary antibodies at 4 °C overnight (the antibody dilution ratio was 1:200). Then, the cells were incubated with a secondary antibody (goat anti-rabbit IgG H&L, 1:500, Cy5/Cy3; Servicebio, Wuhan, China) at 37 °C for 1 h. After washing with PBS, the second-purpose protein antibody was incubated (the antibody dilution ratio was 1:200), and the steps were repeated for bovine serum albumin blocking, followed by incubation with another fluorescent secondary antibody (goat anti-rabbit IgG H&L, 1:500, CoraLite594-conjugated; Proteintech, Wuhan, China). After the nuclei were stained with 4′,6-diamidino-2-phenylindole (DAPI) (Solarbio, Beijing, China), the coverslips were covered with a slide containing an anti-fluorescence quencher (Solarbio, Beijing, China). After panoramic scanning, the fluorescent-labeled cell creeps or tissue sections were observed and photographed with a microscope (Olympus Corporation or 3DHISTECH) using the corresponding software.

### Phalloidin staining

The previous step was the same as that for cellular immunofluorescence before treatment with 0.5% Triton X-100. The cells were washed with PBS and incubated with 100 nM phalloidin-tetramethylrhodamine isothiocyanate (Maokang Biotechnology, Shanghai, China) in PBS (containing 0.1% bovine serum albumin) at room temperature for 30 min. After the cells were washed with PBS to remove excess stain, DAPI was used to restain the nuclei, and the cells were then observed and photographed under a confocal microscope.

### Flow cytometry

Cells in the logarithmic growth phase were seeded into 6-well plates. After the cells were treated with APPswe or APPswe-C for 48 h, they were then collected and fixed with 70% ethanol at 4 °C overnight. Next, the cells were incubated with PI/Triton X-100 for 15 min, and the cell cycle distribution was examined using flow cytometry. Second-generation rat BMSCs (rBMSCs) were collected, and then the cells were suspended in 100 μL of PBS (>10^6^ cells/group). Next, the cells were incubated with FITC-conjugated anti-rat CD45, PE-conjugated anti-rat CD90, and PE-conjugated anti-rat CD29 antibodies at 4 °C for 30 min. The double-negative control group was not treated, and then the samples were tested by flow cytometry.

### Colony-forming unit-fibroblast (CFU-F) assays

Cells were plated in 65 mm petri dishes and transfected with the APPswe or APPswe-C plasmid when they reached 60%–70% confluence, after which the cells were digested and counted. Each group of cells was subsequently cultured at a density of 1 × 10^3^ cells per well in 6-well plates for 8 days, and BMP2 was added to the corresponding group. At the indicated times, the cells were washed three times with PBS and fixed with 4% paraformaldehyde at 37 °C for 10 min. Then, the cells were washed three times with PBS and stained with crystal violet dye for 10 min, and the cells were gently rinsed with running water and air-dried. Under the microscope, a cluster of more than 50 cells was considered an efficient colony.

### Live/dead cell assays

The biocompatibility of the hydrogels was detected using calcein-AM/PI live/dead cell staining kits (Yeasen, Shanghai, China), and the assay was carried out according to the manufacturer's instructions. Primary rBMSCs (approximately 1 × 10^6^ cells) were mixed with 10 μL of 5% GelMA hydrogel and then cross-linked with ultraviolet light for 8–10 second (s) after they were evenly spread into 96-well plates (Fig. S1C). Then, 100 μL of medium containing 10% fetal bovine serum was added to each well, and the cells were cultured for 1, 3, 5, or 7 days. The medium in the 96-well plates was discarded, the plates were washed three times with PBS, 100 μL of working solution (1 × buffer assay buffer: calcein-AM: PI = 1000: 1: 3) was added to each well, and the plates were incubated for 15 min in the incubator. Live (green) and dead (red) cells were visualized by fluorescence microscopy (Nikon Ti2-U).

### MTT assays

The cell viability in the hydrogels was tested by the 3-(4,5-dimethylthiazol-2-yl)-2,5-diphenyl-tetrazolium bromide (MTT) colorimetric assay. The cells were treated and cultured via the same live/dead staining procedure as described above. Then, 5 mg/mL MTT solution (Solarbio, Beijing, China) was added to each well at the specified time points, and the plates were incubated at 37 °C for 4 h. The MTT solution was removed, and 150 μL of dimethyl sulfoxide (BioFROXX, Germany) was added to each well for 15 min. Cell viability was determined by measuring the absorbance at 492 nm using a microcoder, and each group was measured three times.

### Wound healing test

The cells were seeded into a 6-well plate and treated with APPswe or APPswe-C when they reached 60%–70% confluence. A wound was made in the monolayer with a 10 μL pipette tip after 24 h. After washing with PBS, serum-free medium was added, and BMP2 was added to the corresponding groups. The specific wound areas were photographed at 0, 12, or 24 h. The wound healing rate was calculated as follows: (0 h width – 12 h or 24 h width)/0 h width × 100%.

### Transwell migration assay

The cells were treated using the same method as described above, and then the APPswe- or APPswe-C-treated cells were collected and counted. Approximately 20,000 cells per well were seeded into the upper Transwell chamber with 400 μL of serum-free medium. In addition, 500 μL of medium containing 10% fetal bovine serum and supplemented with BMP2 was added to the wells. The cells were cultured for 24 h, fixed with 4% paraformaldehyde, and stained with crystal violet dye for 10 min. Finally, the sections were observed and photographed under a microscope, and the data were analyzed using ImageJ.

### BMP2 release profile

BMP2 (176 μg) was mixed with 200 μL of 5% GelMA hydrogel, the BMP2-hydrogel mixture was incubated in an EP tube containing 1 mL of PBS at 37 °C, and the mixture was agitated at 100 rpm/min. At the indicated time points within 25 days, the solution was aspirated completely and replenished with the same volume of PBS. The cumulative release of BMP2 at a specific time point was quantified using a human BMP2 ELISA kit (Biosharp, Hefei, China), and all operations were carried out according to the manufacturer's instructions. The samples were tested in triplicate.

### Quantitative real-time PCR (qRT-PCR) analysis

High-quality total RNA was extracted using an RNA extraction kit (Accurate Biotechnology, Hunan, China). Two micrograms of total RNA were reverse-transcribed into cDNA with a reverse transcription kit according to the manufacturer's instructions (MCE, New Jersey, USA). Next, qRT-PCR was performed using SYBR Green qPCR Master Mix (Bimake, Texas, USA). Relative gene expression was calculated using the 2^−ΔΔCT^ method. The sequences of the primers (Tsingke, Chengdu, China) used are listed in Table 1, and GAPDH was used as a reference for normalization and to quantify the relative expression levels of the target genes. The touchdown cycling program for qRT-PCR was performed as described previously.^36^^,^^37^

**Table 1:** PCR primers.

Primer name	Length	Primer sequence
Runx2	196 bp	Forward:5′ CCAACTTCCTGTGCTCCGTG 3′
		Reverse:5′ TCGTTGAACCTGGCTACTTGG 3′
OPN	199 bp	Forward:5′ ACACTTTCACTCCAATCGTCC 3′
		Reverse:5′ TGCCCTTTCCGTTGTTGTCC 3′
OCN	199 bp	Forward:5′ TCTGACAAAGCCTTCATGTCC 3′
		Reverse:5 ′AAATAGTGATACCGTAGATGCG 3′
OSX	132 bp	Forward:5′ GGGAGCAGAGTGCCAAGA 3′
		Reverse:5 ′ TACTCCTGGCGCATAGGG 3′
ALP	117 bp	Forward:5′ TGCCCTGAAACTCCAAAAGC 3′
		Reverse:5′ TGTAGCTGGCCCTTAAGGATTC 3′
Hey1	123 bp	Forward:5′ TATCGGAGTTTGGGGTTTCG 3′
		Reverse:5′ TGCGTAGTTGTTGAGATGGGAG 3′
Hes1	174 bp	Forward:5′ GTCTAAGCCAACTGAAAACACTG 3′
		Reverse:5 ′ GGTATTTCCCCAACACGCTC 3′
GAPDH	117 bp	Forward:5′ GACATCAAGAAGGTAATGAAGC 3′
		Reverse:5′ GAAGGTGGAAGAGTGGGAGTT 3′

### Western blotting

Cells were collected and lysed in RIPA lysis buffer containing 1% protease/phosphatase inhibitors (Roche, Basel, Switzerland). The cell lysates were centrifuged at 13,000 *g* for 15 min, and the protein concentration was measured with a BCA protein assay kit (Beyotime, Shanghai, China). Equivalent amounts of protein were separated by SDS‒PAGE, and the bands were then transferred to polyvinylidene difluoride (PVDF) membranes. The PVDF membranes were blocked with 5% bovine serum albumin in Tris-buffered saline with Tween 20 at 37 °C for 2 h and then incubated with primary antibodies at 4 °C overnight (the antibody dilution ratio was 1:500). Afterwards, the membranes were washed with Tris-buffered saline with Tween 20 and incubated with horseradish peroxidase-conjugated secondary antibodies (the antibody dilution ratio was 1:5000; Jackson ImmunoResearch, USA) at 37 °C for 1 h. Specific bands were visualized with an enhanced chemiluminescence kit (Millipore, USA) and imaged with a gel imaging system.

### Establishment of the rat skull critical point defect model

The animal experimental groups were as follows: blank, 5% GelMA, 5% GelMA + rBMSCs, 5% GelMA + rBMSCs + BMP2, 5% GelMA + rBMSCs-APP + BMP2, and 5% GelMA + rBMSCs-APP-C + BMP2 (referred to as the blank, GelMA, rBMSC, BMP2, APPswe, and APPswe-C groups, respectively), and there were four rats in each group. Sprague–Dawley rats were obtained from the Animal Center of Chongqing Medical University (Chongqing, China). After weighing, the rats were anesthetized by intraperitoneal injection of 2.5% pentobarbital sodium (40 mg/kg). The defect was prepared with a 5 mm dental drill. The rBMSCs treated with APPswe or APPswe-C for 24 h were collected (approximately 3 million cells were used in each defect), after which the cells were mixed with 5% GelMA hydrogel (10 μL/group) or BMP2 (10 μg/group). The mixed solution was then used to fill the defect layer by layer under the condition of ultraviolet crosslinking. After the incision was sutured and disinfected, the rats were kept for six weeks, after which their skulls were recovered.

### Micro-CT analysis and histological procedure

Excess soft tissue was removed from the recovered skulls, which were then fixed with 4% paraformaldehyde. A vivaCT 40 microCT system (Scanco Medical) was used to acquire data on bone formation in the defect area, consisting of bone mineral density, bone volume, relative bone volume, trabecular thickness, trabecular number, and trabecular separation values, and the corresponding data were statistically analyzed. Next, the skulls were immersed in EDTA decalcification solution (Solarbio, Beijing, China) and decalcified for 2 weeks at room temperature on a shaking table at 100 rpm/min. The samples were dehydrated through an alcohol gradient and embedded in paraffin blocks. Sections of the embedded specimens were stained with hematoxylin and eosin, Alcian blue, or Masson's trichrome according to the manufacturer's recommendations (Solarbio, Beijing, China). Immunohistochemistry was performed using a ZSGB-BIO kit (ZSGB Biotechnology, Beijing, China) according to the manufacturer's instructions (the antibody dilution ratio was 1:200, and the samples were incubated at 4 °C overnight). After panoramic scanning, the stained tissue sections were observed and photographed using a microscope (Olympus Corporation or 3DHISTECH) and the corresponding software.

### Statistical analysis

Statistical analysis was performed using GraphPad Prism 9.3.1 (GraphPad Software, USA). Each experiment was done in triplicate and repeated at least three times, and all results are expressed as mean ± standard deviation. One-way analysis of variance and two-way analysis of variance were used to compare the differences between groups. The differences were considered statistically significant at *p* < 0.05.

## Results

### Relationship between APP and the osteogenic differentiation, proliferation, and migration of MSCs

**Figure 1:**
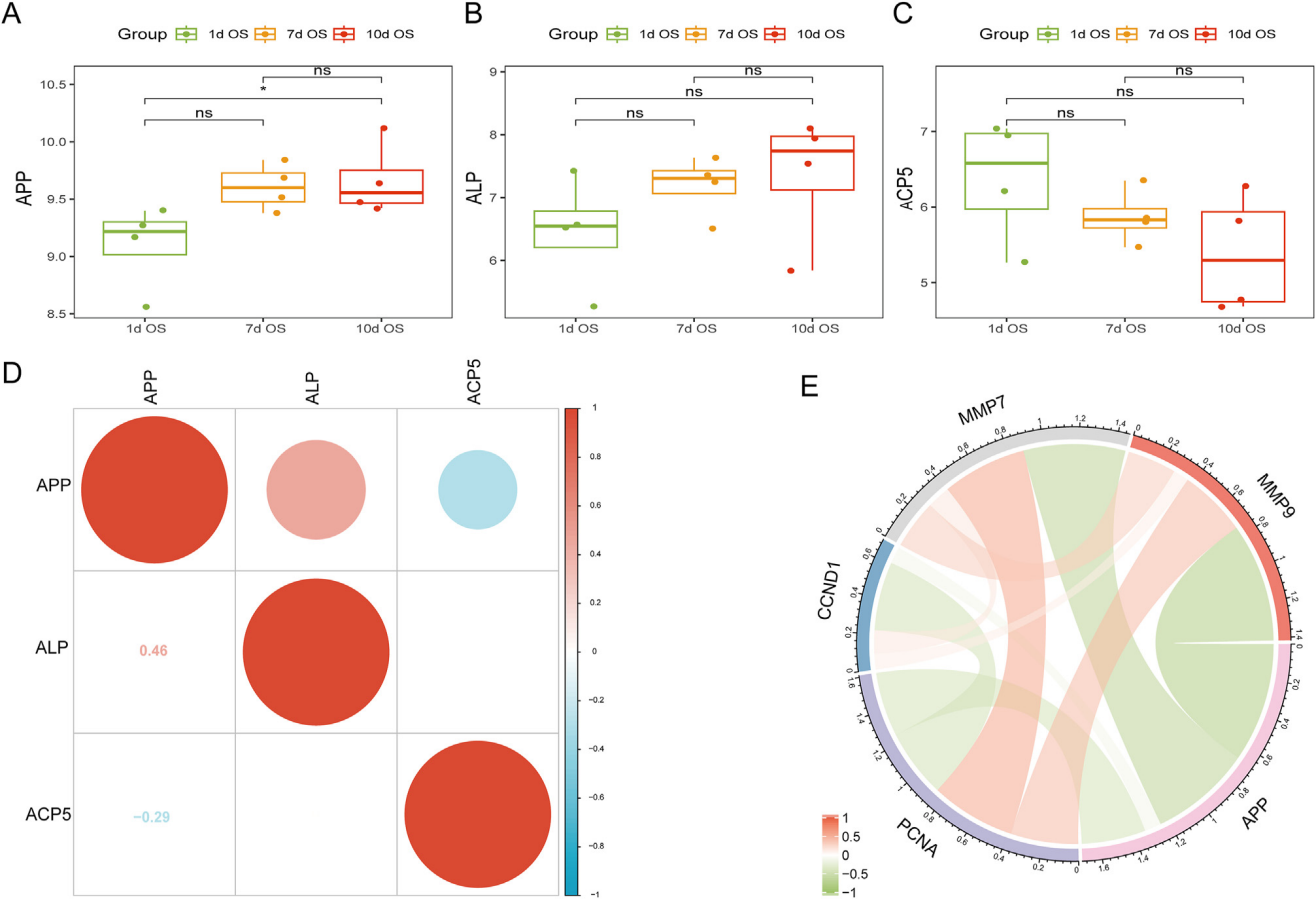
Bioinformatics was used to preliminarily explore the molecular mechanism of APPswe in osteogenic transformation. **(A)** APP expression at different time points during the osteogenic differentiation of MSCs. **(B)** ALP expression at different time points during the osteogenic differentiation of MSCs. **(C)** ACP5 expression at different time points during the osteogenic differentiation of MSCs. **(D)** Analysis of the correlations between the expression of APP and those of ALP and ACP5. **(E)** Correlation analysis between APP and the proliferation genes PCNA and CCND1 and the migration genes MMP7 and MMP9. ∗*p* < 0.05, ∗∗*p* < 0.01, ∗∗∗*p* < 0.001; ns, no significant difference. APP, amyloid precursor protein; APPswe, Swedish mutant APP; MSCs, mesenchymal stem cells; ALP, alkaline phosphatase; ACP5, acid phosphatase 5; PCNA, proliferating cell nuclear antigen; CCND1, cyclin D1; MMP7/9, matrix metallopeptidase 7/9; OS, osteogenesis.

Currently, there is ongoing debate regarding the role of APP in osteogenic differentiation. We evaluated the natural expression of genes related to the osteogenic differentiation of stem cells by analyzing gene expression data from the GEO database. The results showed that the expression of APP increased gradually during the osteogenic differentiation of MSCs, and the difference was statistically significant on day 10 compared with day 1 (Fig. 1A). During the osteogenic differentiation of MSCs, ALP expression also showed a gradually increasing trend, while the expression of the osteoclast activity gene ACP5 showed the opposite trend (Fig. 1B and C), although there was no significant difference between the different time points. Further examination of the associations between APP and ALP, as well as between APP and ACP5, revealed that the correlation coefficient between APP and ALP was 0.46, indicating a positive correlation. Conversely, the correlation coefficient between APP and ACP5 was −0.29, indicating a negative correlation (Fig. 1D). Finally, the correlation between APP and genes associated with cell proliferation and migration was analyzed. There were negative correlations between APP and the proliferation-related genes PCNA and CCND1, and negative correlations were also observed between APP and the migration-related genes MMP7 and MMP9 (Fig. 1E). These findings suggest that APP may have a positive regulatory effect on the osteogenic differentiation process of MSCs, but it may inhibit the proliferation and migration of MSCs, which is consistent with our initial hypothesis.

### APPswe promotes BMP2-induced osteogenic differentiation of MSCs

**Figure 2:**
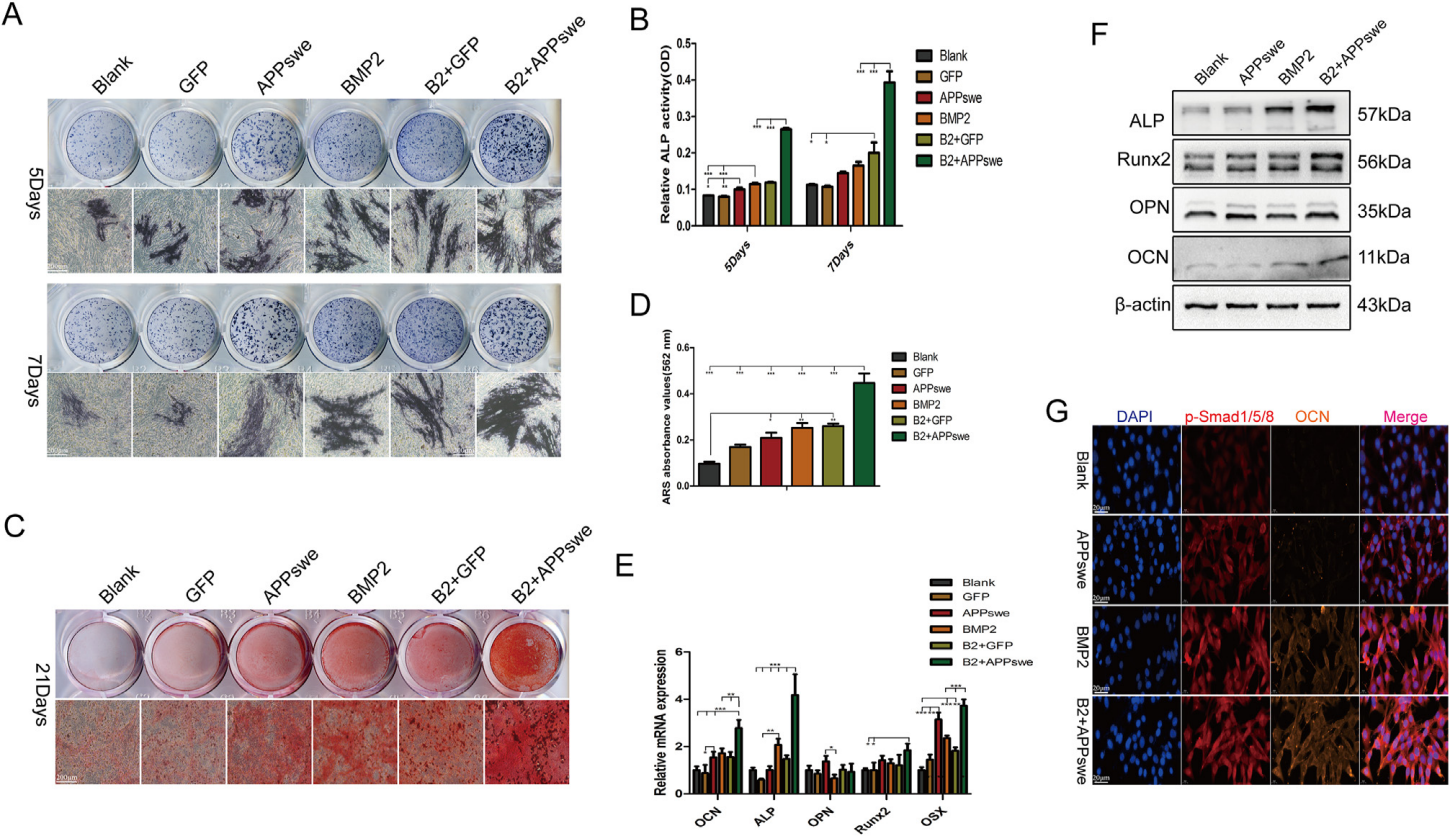
Effect of APPswe on the osteogenic differentiation of MSCs induced by BMP2. **(A, B)** ALP staining and activity detection were performed after treating cells with APPswe or BMP2 for 5 days or 7 days. **(C, D)** ARS staining and semiquantitative analysis were used to detect calcium nodule formation after cells were treated with APPswe or BMP2 for 21 days. **(E)** The mRNA expression levels of genes related to osteogenic differentiation were measured by quantitative real-time PCR after cells were treated with APPswe or BMP2 for 48 h. **(F)** Western blot analysis was performed to detect the expression levels of osteogenic differentiation-related proteins after cells were treated with APPswe or BMP2 for 48 h. **(G)** The expression levels of the osteogenic differentiation proteins p-Smad1/5/8 and OCN in the different treatment groups were detected by cell immunofluorescence. MSCs were treated with APPswe for 24 h and then with BMP2 for 48 h to detect the expression of related proteins or mRNAs. *n* = 3; ∗*p* < 0.05, ∗∗*p* < 0.01, ∗∗∗*p* < 0.001. APPswe, Swedish mutant amyloid precursor protein; MSC, mesenchymal stem cell; ALP, alkaline phosphatase; BMP2, bone morphogenetic protein 2; OCN, osteocalcin.

We analyzed the effect of APPswe on the osteogenic differentiation of MSCs induced by BMP2 (500 ng/mL) to verify the relationship between APPswe and the osteogenic differentiation of MSCs. ALP detection revealed that APPswe significantly promoted the production of ALP (Fig. 2A and B), and ARS staining and semiquantitative analysis also revealed that calcium salt deposition increased significantly in the APPswe treatment group (Fig. 2C and D). Further, qRT-PCR revealed that the expression levels of the osteogenic differentiation-related genes *ALP*, *Runx2*, *OSX*, and *OCN* were most significantly increased in the group treated with BMP2 and APPswe (Fig. 2E). The protein expression levels detected were consistent with the aforementioned results (Fig. 2F). Furthermore, the cellular immunofluorescence results indicated significant increases in the levels of p-Smad1/5/8 and OCN in the APPswe treatment group (Fig. 2G). The above results suggest that APPswe may have a positive regulatory effect on the osteogenic differentiation of MSCs.

### APPswe inhibits the proliferation and migration of MSCs

**Figure 3:**
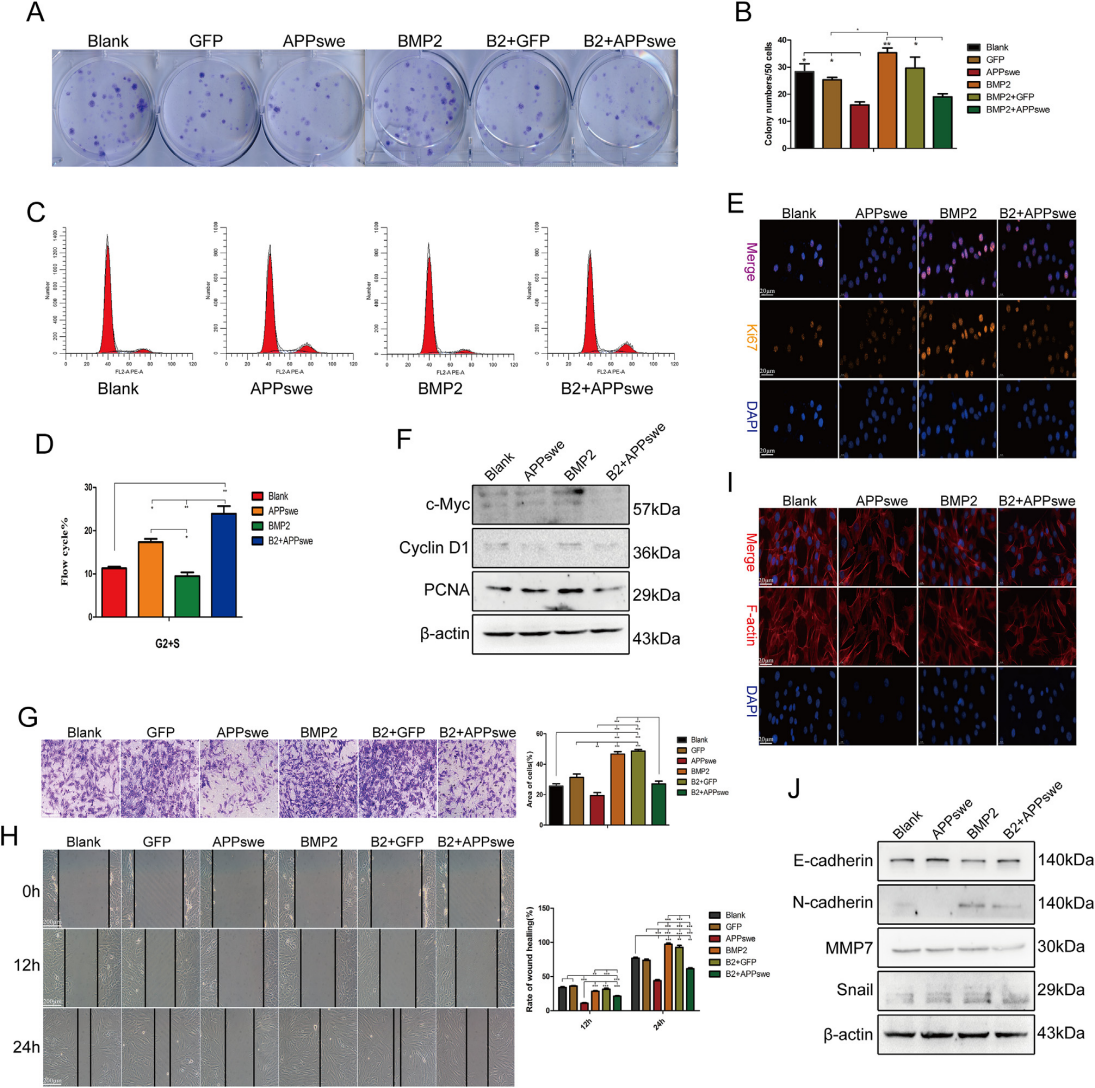
APPswe inhibits the self-renewal, proliferation, and migration of MSCs. **(A, B)** The effect of APPswe on the self-renewal ability of MSCs was detected by colony-forming unit-fibroblast assays. **(C, D)** Cell cycle changes in MSCs treated with APPswe were detected by flow cytometry. **(E)** The expression of the proliferation-related gene Ki67 in different groups was detected by cell immunofluorescence. **(F)** The expression levels of proliferation-related proteins in MSCs after treatment with APPswe were detected by western blotting. **(G)** Transwell assays were used to detect the effect of APPswe treatment on the cell migration ability of MSCs. **(H)** The effect of APPswe on MSC migration was detected by a wound healing test. **(I)** The expression levels of F-actin in the different treatment groups were detected by FITC-phalloidin staining. **(J)** Western blot analysis was performed to detect the expression levels of migration-related proteins in the different groups. *n* = 3; ∗*p* < 0.05; ∗∗*p* < 0.01, ∗∗∗*p* < 0.001. APPswe, Swedish mutant amyloid precursor protein; MSC, mesenchymal stem cell.

In addition to the osteogenic differentiation ability and osteoclast activity of MSCs, the self-renewal, proliferation, and migration abilities of MSCs are also involved in the regulation of bone formation. The results of the CFU-F assays revealed that the APPswe group exhibited fewer and smaller colonies than the BMP2 group (Fig. 3A and B). The cell cycle results showed that the cells were partially arrested in the S/G2 phase after treatment with APPswe (Fig. 3C and D), and the cellular immunofluorescence results showed that BMP2 promoted the expression of Ki67, while APPswe inhibited it (Fig. 3E). Furthermore, the expression levels of proliferation-related proteins were significantly decreased in the APPswe group (Fig. 3F). In addition, the results of Transwell and wound healing assays showed that BMP2 promoted the migration of MSCs,^38^ while APPswe inhibited this migration (Fig. 3G and H). After treatment with APPswe, the cytoskeleton morphology was significantly disrupted, and the expression of F-actin was significantly reduced (Fig. 3I). Moreover, the expression levels of migration-related proteins were significantly decreased in the APPswe group, while the expression of E-cadherin was significantly increased (Fig. 3J). These findings suggest that APPswe can promote the osteogenic differentiation of MSCs while simultaneously suppressing their self-renewal, proliferation, and migration abilities.

### AICD plays an important role in promoting osteogenic differentiation

**Figure 4:**
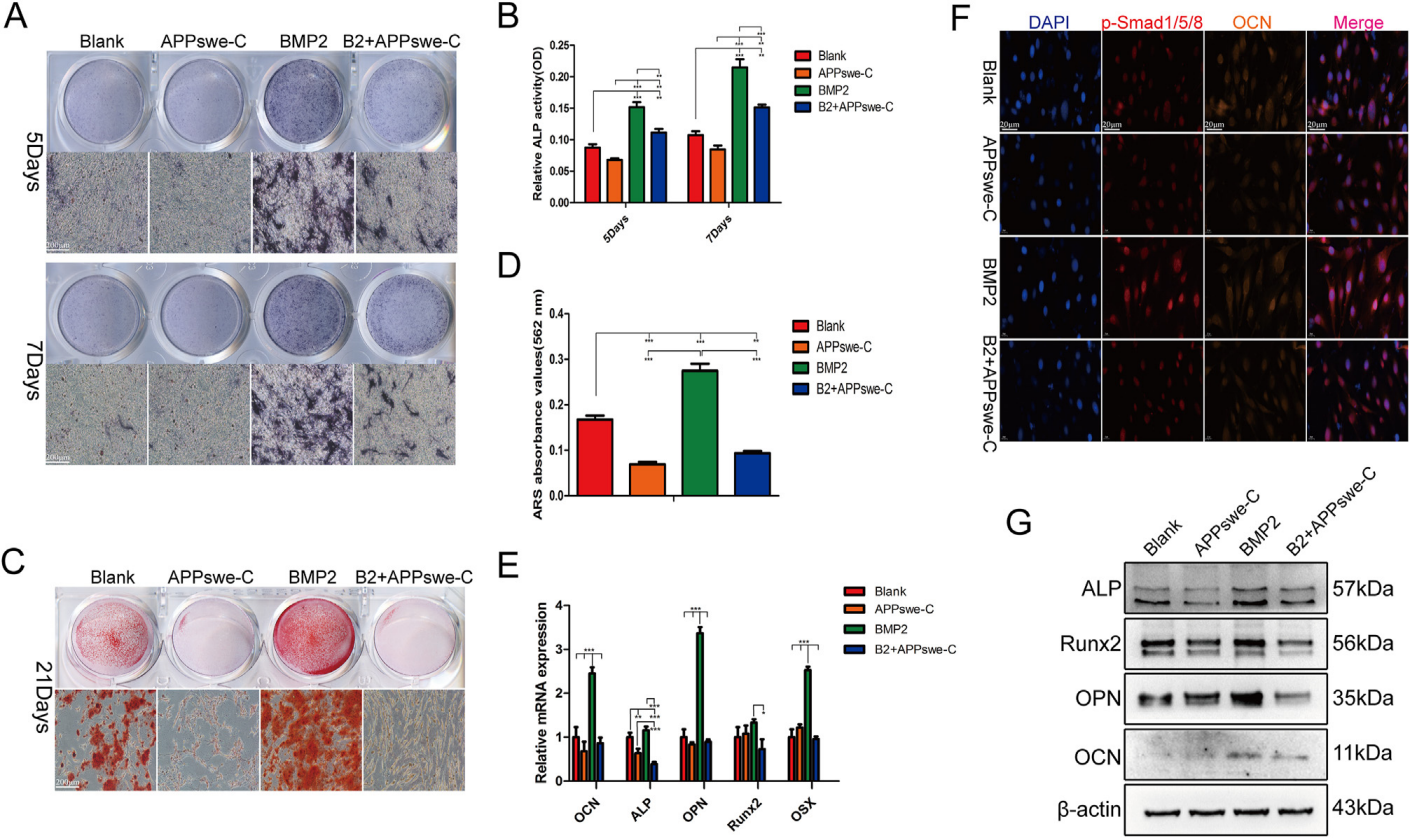
AICD plays a crucial role in promoting the BMP2-induced osteogenic differentiation of MSCs. **(A, B)** ALP staining and activity detection were performed after treating cells with APPswe-C or BMP2 for 5 and 7 days. **(C, D)** ARS staining and semiquantitative analysis were used to detect calcium nodule formation in cells treated with APPswe-C or BMP2 for 21 days. **(E)** The mRNA expression levels of genes related to osteogenic differentiation were measured by quantitative real-time PCR after cells were treated with APPswe-C or BMP2 for 48 h. **(F)** The expression levels of the osteogenic differentiation proteins p-Smad1/5/8 and OCN in the different treatment groups were detected by cell immunofluorescence. **(F)** Western blot analysis was performed to detect the expression levels of osteogenic differentiation-related proteins after cells were treated with APPswe or BMP2 for 48 h. MSCs were treated with APPswe-C for 24 h and then with BMP2 for 48 h to detect the expression levels of related proteins or mRNAs. *n* = 3; ∗*p* < 0.05, ∗∗*p* < 0.01, ∗∗∗*p* < 0.001. APPswe, Swedish mutant amyloid precursor protein; AICD, the intracellular domain of APPswe; BMP2, bone morphogenetic protein 2; MSC, mesenchymal stem cell; ALP, alkaline phosphatase; APPswe-C, APPswe without an intracellular segment; OCN, osteocalcin.

To explore the mechanism by which APPswe regulates the osteogenic differentiation of MSCs, further experiments were conducted to confirm the effect of APPswe-C on the osteogenic differentiation of MSCs. The results of ALP staining and activity detection (Fig. 4A and B) as well as ARS staining (Fig. 4C and D) demonstrated that APPswe-C significantly inhibited the osteogenic differentiation of MSCs induced by BMP2. Furthermore, both the mRNA and protein expression levels of osteogenesis-related genes were significantly suppressed in the APPswe-C-treated group (Fig. 4E–G). The cellular immunofluorescence results also revealed notable reductions in the expression of osteogenic differentiation-related proteins after treatment with APPswe-C (Fig. 4F). These findings suggest that AICD may play an important role in promoting the osteogenic differentiation of MSCs, but the extracellular fragment Aβ appears to have an inhibitory effect on the osteogenic differentiation of MSCs.^21^^,^^39^

### APPswe-C inhibits the proliferation and migration of MSCs

**Figure 5:**
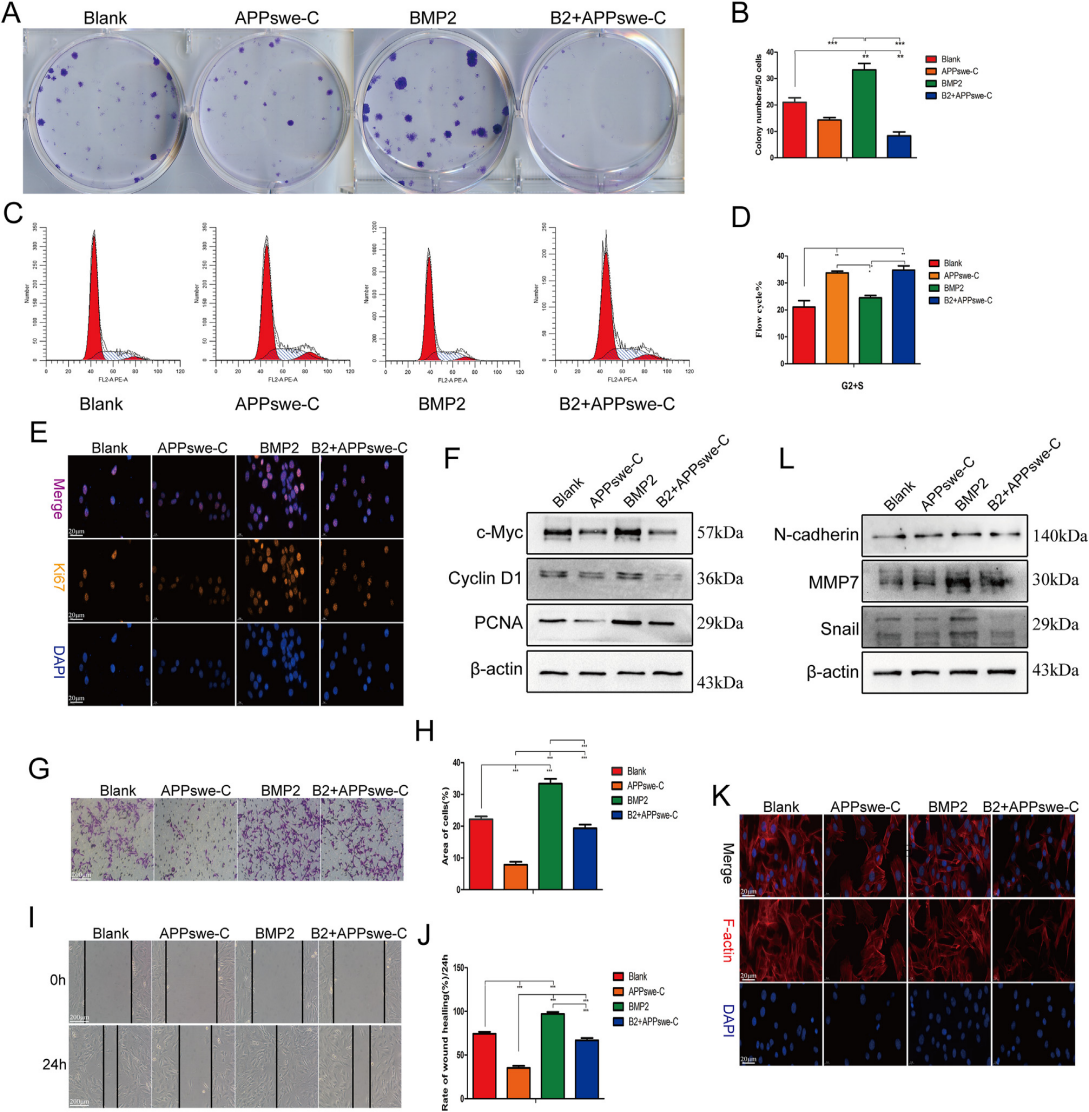
Effects of APPswe-C on the proliferation and migration of MSCs. **(A, B)** The effect of APPswe-C on the self-renewal of MSCs was detected by a colony-forming unit-fibroblast assay. **(C, D)** The effect of APPswe-C on the cell cycle was detected by flow cytometry. **(E)** The expression of the proliferation gene Ki67 was detected by cellular immunofluorescence. **(F)** The expression levels of proliferation-related proteins after APPswe-C treatment were detected by western blotting. **(G, H)** The cell migration of each group after APPswe-C treatment was detected by Transwell assay. **(I, J)** The effect of APPswe-C treatment on cell migration was detected by a wound healing test. **(K)** The expression of F-actin in the cells of each group after APPswe-C treatment was detected by FITC-phalloidin staining. **(L)** The expression levels of cell migration-related proteins were detected by western blot. MSCs were treated with APPswe-C for 24 h and then with BMP2 for 48 h to detect the expression levels of related proteins. *n* = 3; ∗*p* < 0.05, ∗∗*p* < 0.01, ∗∗∗*p* < 0.001. APPswe-C, Swedish mutant amyloid precursor protein without an intracellular segment; MSC, mesenchymal stem cell; BMP2, bone morphogenetic protein 2.

To investigate the potential impact of APPswe-C on the proliferation and migration of MSCs, we analyzed alterations in the cell cycle, proliferation, and migration abilities of MSCs after treatment with APPswe-C. The results of the CFU-F assay showed that the number and size of cell colonies in the APPswe-C treatment group were significantly lower (Fig. 5A and B). The cell cycle was partially blocked in the S/G2 phase in the APPswe treatment group (Fig. 5C and D). Further analysis of the cell immunofluorescence results revealed that the expression of Ki67 was significantly decreased in the APPswe-C treatment group (Fig. 5E). Furthermore, the expression of cell proliferation-related proteins such as c-Myc, PCNA, and CCND1 decreased significantly after treatment with APPswe-C (Fig. 5F). These results suggest that the inhibitory effect of APPswe on MSC proliferation may be caused by Aβ.^14^ Given the confirmation from previous findings that APPswe can inhibit MSC migration, we further analyzed the role of APPswe-C in this process through a series of experiments. First, the results of the wound healing test (Fig. 5G and H) and Transwell assay (Fig. 5I and J) demonstrated that APPswe-C significantly inhibited MSC migration. Phalloidin staining revealed a notable disruption in the morphology of the cytoskeleton and a decrease in F-actin expression following treatment with APPswe-C (Fig. 5K). These findings align with previous observations made on MSCs treated with APPswe. In addition, the results revealed that APPswe-C significantly suppressed the expression of migration-related proteins in MSCs (Fig. 5L). These results suggest that the negative effect of APPswe on MSC proliferation and migration may be closely related to its extracellular fragment.^25^

### APPswe affects the osteogenic differentiation and proliferation of MSCs by regulating the Notch signaling

**Figure 6:**
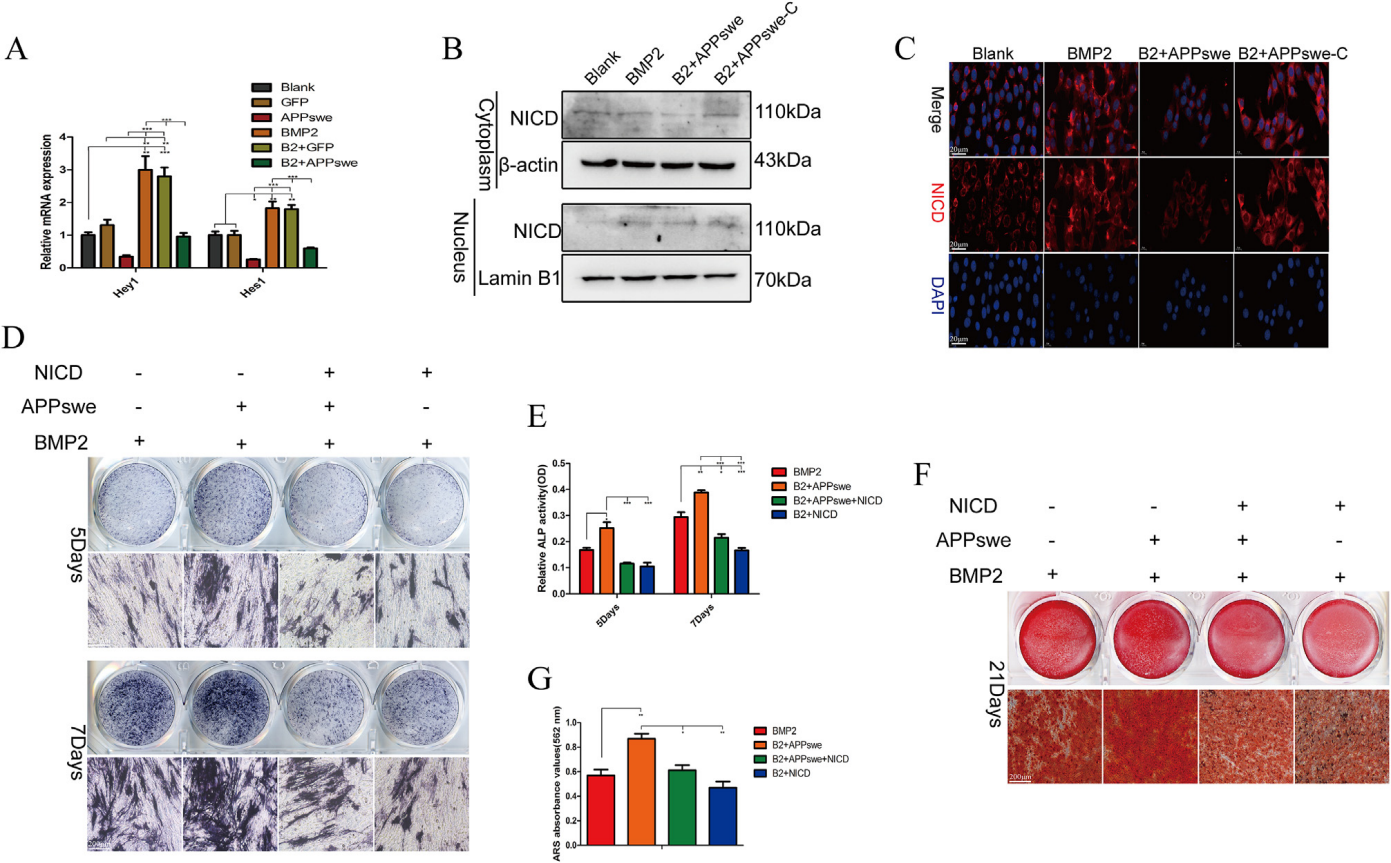
APPswe affects the osteogenic differentiation and proliferation of MSCs by regulating the Notch signaling. **(A)** The mRNA expression levels of *Hey1* and *Hes1* in each treatment group were measured by quantitative real-time PCR. **(B)** Western blot analysis of NICD expression in the cytoplasm and nucleus in each treatment group. **(C)** The expression of NICD in each treatment group was detected by cellular immunofluorescence. **(D, E)** ALP staining and activity detection were performed in each treatment group. **(F, G)** The formation of calcium nodules in each treatment group was analyzed by ARS staining and semiquantitative analysis. MSCs were treated with APPswe or APPswe-C for 24 h and then with BMP2 for 48 h to detect the expression levels of related proteins. *n* = 3; ∗*p* < 0.05, ∗∗*p* < 0.01, ∗∗∗*p* < 0.001. APPswe, Swedish mutant amyloid precursor protein; APPswe-C, APPswe without an intracellular segment; MSC, mesenchymal stem cell; ALP, alkaline phosphatase; BMP2, bone morphogenetic protein 2; NICD, the Notch intracellular domain.

Numerous prior investigations have shown that the Notch pathway can impede the osteogenic differentiation of MSCs while preserving their stem cell characteristics. Additionally, certain studies have addressed the interplay between AICD and NICD.^31^^,^^35^^,^^40^^,^^41^ Therefore, we speculate that APPswe may interact with the key transcription factor NICD of the Notch pathway through AICD to affect Notch pathway activity, thereby promoting the osteogenic differentiation of MSCs while concurrently suppressing their proliferation. First, APPswe significantly inhibited the mRNA expression of *Hey1* and *Hes1*, which are downstream target genes of the Notch pathway (Fig. 6A). Western blot (Fig. 6B) and cellular immunofluorescence (Fig. 6C) results showed that NICD expression in the cytoplasm was significantly decreased in the APPswe treatment group, and its expression in the nucleus was also decreased, while NICD expression in the APPswe-C treatment group did not significantly change. To clarify the role of NCID in promoting the osteogenic differentiation of MSCs, we simultaneously transfected APPswe-treated cells with Ad-NICD. The results of ALP staining and activity detection (Fig. 6D and E), as well as ARS staining (Fig. 6F and G), demonstrated that the up-regulation of NICD effectively counteracted the ability of APPswe to promote the osteogenic differentiation of MSCs. In addition, our previous findings revealed that overexpression of the NICD-activated Notch pathway could promote the self-renewal and proliferation of MSCs but inhibit the osteogenic differentiation of MSCs induced by BMP9.^42^ These results suggest that APPswe may inhibit the cytoplasmic stability and nuclear translocation of the transcription factor NICD through AICD.

### Biocompatibility and protein slow-release properties of the GelMA hydrogel

**Figure 7:**
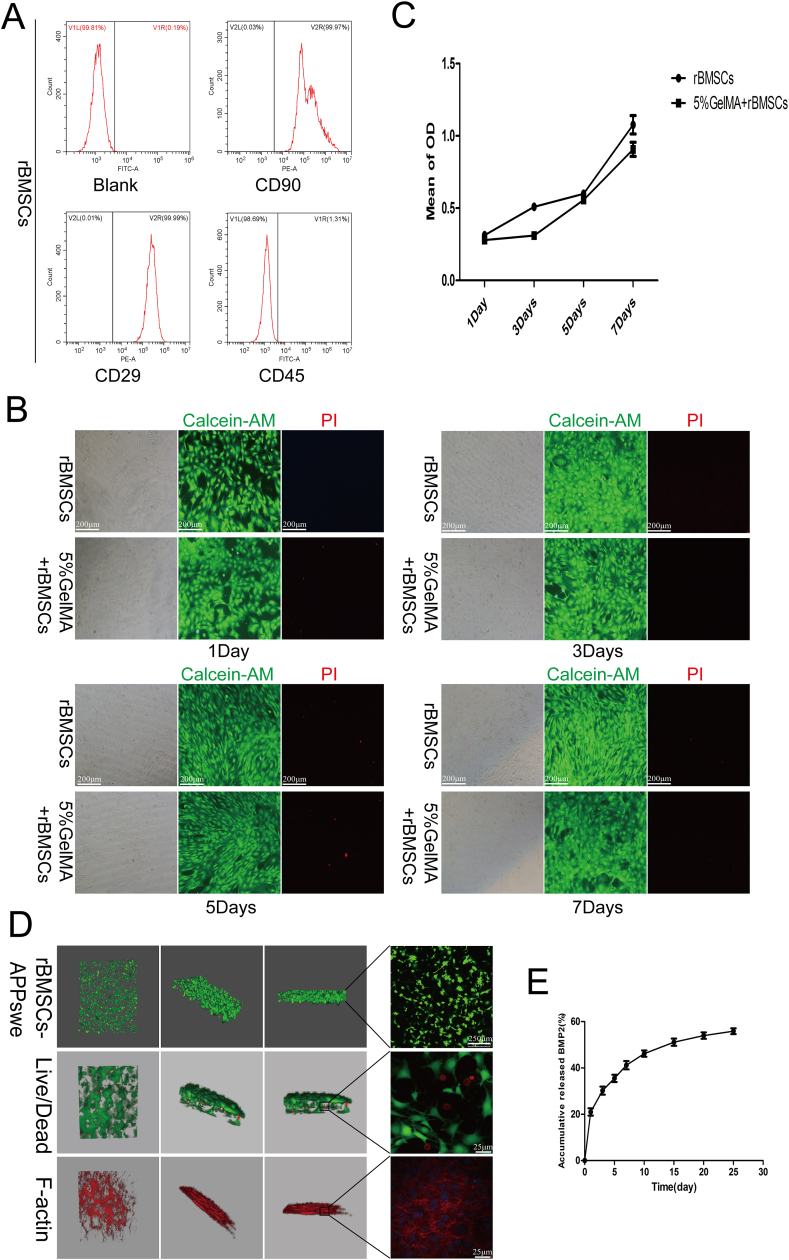
Biocompatibility and protein release properties of the GelMA hydrogel. **(A)** Flow cytometry was used to detect rBMSCs extracted via the adherent method. **(B)** The rBMSCs cultured in GelMA hydrogel for 1, 3, 5, and 7 days were detected by live and dead staining. **(C)** MTT was used to detect the growth of rBMSCs cultured in GelMA hydrogels at different time points. **(D)** Confocal laser scanning was used to detect the specific cell morphology, growth, and extension of the rBMSCs in the GelMA hydrogel. **(E)** ELISA was used to measure the release of BMP2 in the GelMA hydrogel at different time points. APPswe-treated rBMSCs were cultured for 24 h and then collected. *n* = 3; ∗*p* < 0.05, ∗∗*p* < 0.01, ∗∗∗*p* < 0.001. APPswe, Swedish mutant amyloid precursor protein; rBMSC, rat bone mesenchymal stem cell; BMP2, bone morphogenetic protein 2; GelMA, gelatin methacryloyl.

To conduct a more comprehensive investigation into the impact of APPswe on the osteogenic differentiation of MSCs, we devised an *in vivo* experiment utilizing a rat calvarial defect model. We utilized a 5% GelMA hydrogel material to encapsulate APPswe- or APPswe-C-treated rBMSCs and BMP2. This approach allowed us to create a composite scaffold material that could provide scaffold support, seed cells, and osteogenic activity factors at the site of the calvarial defect to determine the bone formation of each group *in vivo*. The results of flow cytometry revealed that the expression levels of the positive markers CD90 and CD29 were significantly high, whereas the negative marker CD45 exhibited weak positivity on the cells isolated from SD rats (Fig. 7A). The live/dead staining and MTT assays revealed that the rBMSCs in the 5% GelMA hydrogel group exhibited normal growth compared with those in the blank group at 1, 3, 5, and 7 d (Fig. 7B and C). Furthermore, the results of confocal laser scanning demonstrated that APPswe-treated rBMSCs were able to grow in the hydrogel material without any abnormalities (Fig. 7D). Moreover, live/dead staining and FITC-phalloidin staining revealed that the rBMSCs were able to survive, adhere, and spread in the hydrogel material (Fig. 7D). The release curve showed that BMP2 was released rapidly on the first day and then slowly and evenly for the next 24 days, indicating that the hydrogel can maintain the activity of the protein while having sustained release characteristics (Fig. 7E). The above results fully demonstrated that the 5% GelMA hydrogel had good biocompatibility, the rBMSCs could grow normally in the hydrogel, and the hydrogel could maintain the activity and sustain the release of BMP2.

### APPswe promotes skull defect repair *in vivo*

**Figure 8:**
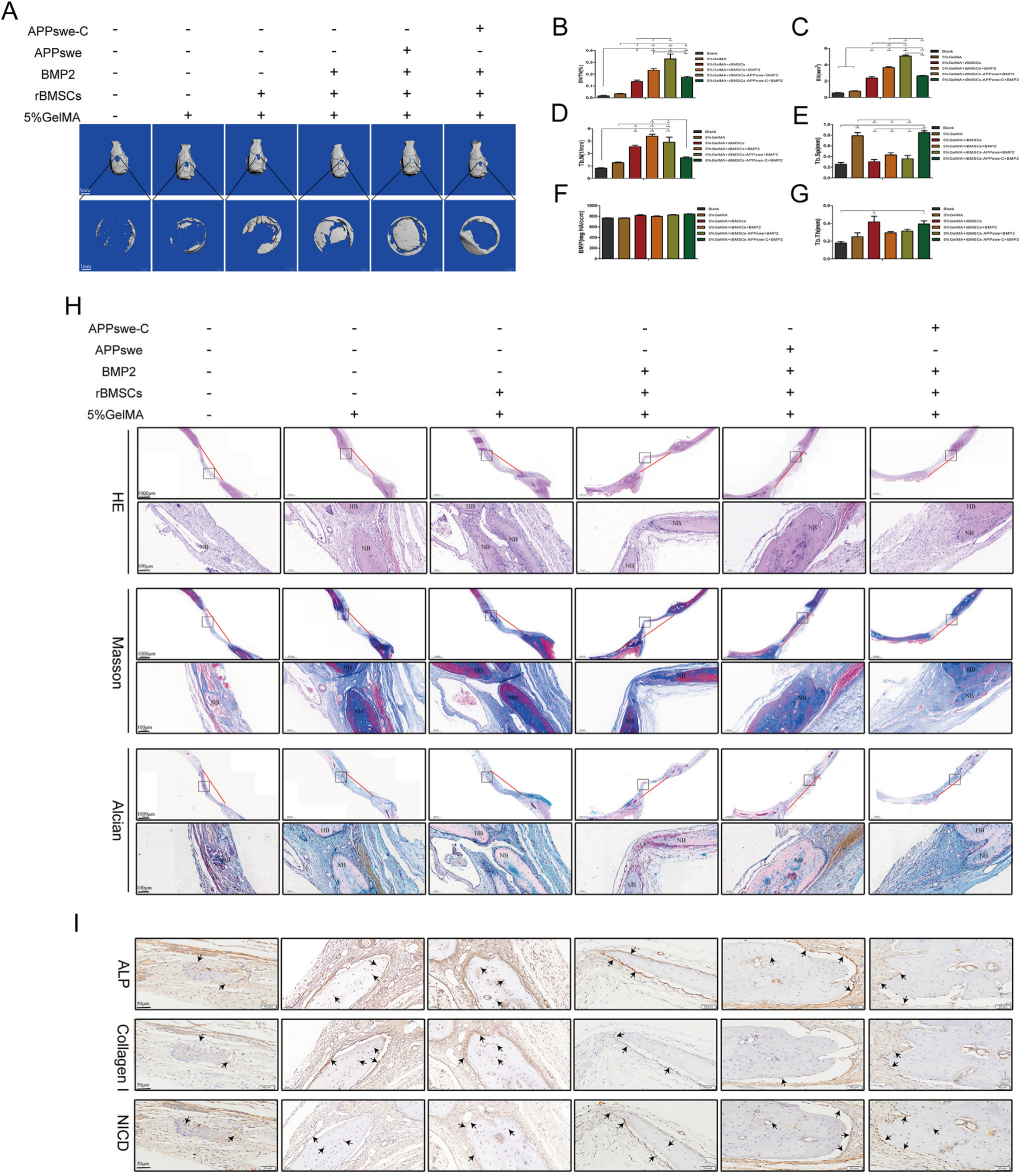
Effects of APPswe on BMP2-induced bone formation and repair in rBMSCs *in vivo.***(A)** Six weeks after surgery, the rat skulls were recovered for micro-CT analysis, and representative images were shown. **(B**–**G)** Histomorphometric analysis of structural bone parameters. Bone volume (BV, mm^3^), relative bone volume (BV/total volume, %), trabecular number (Tb.N, 1/mm), trabecular separation (Tb.Sp, mm), bone mineral density (BMD, mg HA/ccm), and trabecular thickness (Tb.Th, mm) were calculated based on the micro-CT scanning data. **(H)** Representative hematoxylin-eosin staining, Masson's trichrome staining, and Alcian blue staining of each group are shown. **(I)** The expression levels of ALP, collagen I, and NICD were confirmed by immunohistochemical staining. Representative images of each group are shown. The red line indicates the location and width of the skull defect in each group. The black box in the tissue staining image is the locally representative region of the lower-magnification image. The black arrows indicate positive protein expression in new bone. *n* = 3; ∗*p* < 0.05, ∗∗*p* < 0.01, ∗∗∗*p* < 0.001. APPswe, Swedish mutant amyloid precursor protein; rBMSC, rat bone mesenchymal stem cell; ALP, alkaline phosphatase; NICD, the Notch intracellular domain; NB, new bone; HB, host bone.

We subsequently employed a rat skull defect model to verify the effects of different treatments on bone formation *in vivo*. The results specifically show the process of filling rat skull defects with mixed materials composed of rBMSCs-APPswe/APPswe-C, BMP2, and 5% GelMA (Fig. S1D) and various forms of GelMA (Fig. S1E). Next, the effectiveness of the transfection of the rBMSCs with the adenovirus APPswe was detected by fluorescence microscopy (Fig. S1F). After filling the skull defect of 5 mm with materials in each treatment group, the skulls were cross-linked with 365 nm ultraviolet light and were then recovered after 6 weeks. The results of micro-CT reconstruction (Fig. 8A) indicated the absence of bone formation in the skull defects within the blank group, and a limited quantity of bone tissue was observed to have formed at the site of the defects in the GelMA group. In the rBMSC group, there was no large amount of bone tissue formation, which may be related to the lack of inducible factors for osteogenic activity. Compared with the pretreatment group, the BMP2 group exhibited a significant increase in bone tissue generation at the defect sites, which highlighted the important role of osteogenic active factors in bone formation. Bone formation in the APPswe group was significantly greater than that in the other groups, while bone formation in the APPswe-C group was significantly less than that in the APPswe and BMP2 groups, consistent with the results of the *in vitro* experiments. Static histomorphometric analysis revealed that the bone volume of the APPswe group was significantly greater than those of the other groups, and the bone volume of the APPswe-C group was significantly less than those of the APPswe and BMP2 groups (Fig. 8B and C). The effect in the rBMSC group was also significantly greater than those in the control and GelMA groups (Fig. 8B and C), indicating that seed cells and osteogenic active factors are important for bone formation. The numbers of bone trabeculae in the BMP2 and APPswe groups were the greatest, while the number of bone trabeculae in the APPswe-C group was significantly reduced (Fig. 8D). The levels of bone trabecular separation in the GelMA and APPswe-C groups were the greatest, but there was no significant difference between the other groups (Fig. 8E). However, no significant difference was observed in terms of bone density or trabecular thickness (Fig. 8F), with only the rBMSC and APPswe-C groups having slightly greater trabecular thickness than the other groups (Fig. 8G).

The hematoxylin-eosin staining results showed that the number of new bones in the APPswe group was the greatest, followed by that in the BMP2 group, and only small amounts of new bone formation were observed in the other groups. The thickness of new bone in the defect site was also significantly greater in the APPswe group than in the other groups (Fig. 8H). Masson staining analysis revealed that the maturation levels of new bone were significantly greater in the BMP2 and APPswe groups than in the other groups (Fig. 8H). The results of Alcian blue staining showed that there was less cartilage matrix in the new bone of the APPswe group than in the other groups, while there was more cartilage matrix in the new bone in the BMP2 group (Fig. 8H). The expression levels of the osteoblastic markers ALP and collagen I in the APPswe and BMP2 groups were significantly greater than those in the other groups, while the expression of NICD was significantly lower in the APPswe group (Fig. 8I). These findings are consistent with the results of our *in vitro* experiments, supporting the positive effect of APPswe on BMP2-induced osteogenic differentiation of MSCs and indicating that APPswe may exert these effects by regulating the Notch pathway.

## Discussion

AD is a chronic progressive disease, with an average duration of approximately 8 years from diagnosis to onset.^43^ Recent clinical research has revealed that AD not only causes neurological damage but also has significant impacts on various organs and tissues, including bone tissue, and these effects have been observed during the preclinical AD period.^2^^,^^44^ At present, bone loss and osteoporosis have been observed in various animal models of AD, such as hTau mice, APP/PS1 double transgenic mice, and Swedish mutant APP transgenic mice,^17^^,^^18^^,^^45^^,^^46^ indicating that changes in the skeletal system may be related to the pathological basis of AD. However, the effect of APPswe, a pathogenic protein of AD, on the skeletal system and its specific mechanism is still unclear. BMP2, a bone repair factor that has been widely utilized in clinical settings, has demonstrated significant therapeutic efficacy in numerous studies.^47^

In this study, our results showed that APP expression gradually increased during MSC osteogenic differentiation and was positively correlated with the osteogenic differentiation marker ALP but negatively correlated with the osteoclast activity marker ACP5 according to the GEO database. These results are consistent with previous studies showing that APP promotes MSC osteogenic differentiation and increases the expression of OPN in the blood of patients with AD.^13^^,^^32^, ^33^, ^34^ APPswe is one of the most common pathogenic proteins in AD, and we demonstrated that APPswe promotes the osteogenic differentiation of MSCs induced by BMP2, resulting in increased ALP activity and the formation of calcium salt nodules and significantly increased expression levels of the osteogenic differentiation-related proteins OCN, OPN, Runx2, p-Smad1/5/8, and ALP. These results contradict those of several previous studies,^9^^,^^13^ which may be due to the use of different types of APP, Aβ, or cell lines employed. Other studies have indicated that certain Aβ subtypes are similar to Wnt/β-catenin pathway ligands and can activate the Wnt/β-catenin pathway by binding to low-density lipoprotein (LDL) receptor-related protein 5/6 (the receptor) to promote MSCs osteogenic differentiation and inhibit osteoclast activity.^14^ Additionally, the APP-AICD-GSK3β signaling pathway has been shown to promote the differentiation of neural stem cells,^25^^,^^48^, ^49^, ^50^ and we also found that APPswe significantly enhanced the expression of p-GSK 3β (Ser9) and β-catenin in this study (Fig. S1G).

Most studies have focused mainly on the toxic effects of Aβ aggregates in the nervous system, while the functional role of its 6-kDa intracellular fragment, AICD, and its effect are still controversial, and its role in osteogenic differentiation has rarely been reported.^23^ AICD is similar to the Aβ peptide, and it has multiple subtypes, including AICD59, AICD57, AICD51, AICD50, and AICD31,^23^^,^^24^ based on the number of amino acids it contains, due to the presence of different γ-secretase cleavage sites. Some studies have indicated that AICD is involved in regulating the proliferation and differentiation of neural cells.^23^^,^^51^ Therefore, based on the common AICD57 subtype, we designed the APPswe-C overexpression plasmid. We demonstrated that APPswe-C inhibits the osteogenic differentiation of MSCs induced by BMP2, as evidenced by the significantly reduced expression levels of the osteogenic differentiation-related genes OCN, OPN, Runx2, p-Smad1/5/8, and ALP in the APPswe-C-treated group. These results indicate that the extracellular segment of APPswe may inhibit the osteogenic differentiation of MSCs, which is consistent with previous research results.^52^^,^^53^ However, AICD has a promoting effect on MSCs osteogenic differentiation, but the specific mechanism of action needs further study.

The traditional view ignores the effects of MSCs self-renewal, proliferation, and migration on bone formation, and previous studies have not investigated the effects of APPswe on the above functions of MSCs. For these reasons, we also analyzed the effects of APPswe on the proliferation and migration abilities of MSCs. Our results showed that APPswe inhibited MSCs proliferation and migration, and the expression levels of related proteins were also affected. Previous studies have indicated that APPswe can stimulate the proliferation and differentiation of neural stem cells, but some reports have suggested that APPswe inhibits cell proliferation.^25^^,^^49^^,^^51^^,^^54^^,^^55^ To further clarify the role of the extracellular segments of APPswe in regulating the proliferation and migration of MSCs. We found that APPswe-C could simultaneously inhibit the proliferation and migration of MSCs, indicating that the extracellular segment of APPswe plays an important negative role in affecting MSCs proliferation and migration. In addition, the cytoskeletons of MSCs were both significantly disrupted and decreased after APPswe or APPswe-C treatment. Previous studies have also suggested that AICD could interfere with the F-actin ability of cells by affecting mitochondrial energy metabolism.^56^ In addition, AD can cause neuronal aging, which may lead to insufficient activity of age-related bone cells and bone loss,^2^ and this may be the cause of severe bone loss in AD patients.

Previous studies have confirmed that APP or APPswe can affect cell proliferation, differentiation, and other functions by regulating a variety of signaling pathways or oxidative stress.^17^^,^^25^^,^^57^^,^^58^ The Notch pathway plays an important role in the regulation of MSCs differentiation and proliferation.^31^ In addition, previous studies have shown that NICD and AICD have similar cell localizations and that there are interactions between the two proteins.^27^, ^28^, ^29^^,^^59^ Our results showed that APPswe inhibits the expression of *Hey1* and *Hes1* in the Notch pathway and inhibits the expression and nuclear translocation of the transcription factor NICD. NICD overexpression reversed the ability of APPswe to promote the osteogenic differentiation of MSCs. To further determine whether APPswe has the same effect *in vivo*, we used recombinant BMP2 protein as the osteogenic differentiation-inducing factor for MSCs and GelMA hydrogel as the carrier scaffold for loading BMP2- and APPswe- or APPswe-C-treated rBMSCs to form a composite material, and a rat skull defect model was used to analyze the effects of APPswe or APPswe-C on the bone repair and differentiation abilities of the rBMSCs. The biocompatibility of GelMA hydrogels has been validated in this study and many other research models,^60^^,^^61^ and the resulting cells can grow, survive, and maintain the activity and slow release of the recombinant protein BMP2.^62^

The rBMSCs from each group were collected after treatment with APPswe or APPswe-C and then mixed with 10 μg of BMP2 or 10 μL of 5% GelMA hydrogel. The skull specimens were collected 6 weeks after the operation, and the micro-CT and histological results showed that the APPswe group had the highest amounts of newly formed bone and bone trabeculae compared with the APPswe-C-treated group, as well as the highest expression levels of osteogenic differentiation-related proteins. These findings were in line with the outcomes of the *in vitro* cellular experiments and provided additional confirmation of the ability of APPswe to promote the osteogenic differentiation of MSCs.^14^^,^^16^ There was more newly formed bone in the 5% GelMA group than in the control group, although the difference was not significant. A possible explanation for this phenomenon could be attributed to the GelMA hydrogel, which serves as a supportive structure facilitating the infiltration of tissue during the bone regeneration procedure. In the BMP2 group, there were significant increases in newly formed bone, bone trabeculae, and osteogenic differentiation-related proteins compared with those in the BMSC group, which fully demonstrated the essential roles of osteogenic induction factors in bone formation and repair. The thickness of the bone trabeculae only slightly increased in the rBMSC and APPswe-C groups, but the bone trabecular space was most evident in the GelMA and APPswe-C groups, which was consistent with the inhibitory effect of APPswe-C on the osteogenic differentiation of MSCs. However, limited by the small number of clinical patients with AD and experimental conditions, we were unable to observe and analyze the APPswe expression, bone density, proliferation, and osteogenic differentiation ability of BMSCs at different stages in AD patients or AD animal models, as well as the relationships between them. In subsequent experiments, we intend to further verify our hypothesis in AD animal models and analyze the specific mechanism by which the interaction between AICD and NICD affects the osteogenic differentiation of MSCs. Similarly, the extracellular and extracellular segments of APPswe play different roles in regulating the osteogenic differentiation of MSCs, and the specific mechanisms involved are worthy of further investigation.

In conclusion, our study demonstrated that APPswe could affect the expression and nuclear transfer of the transcription factor NICD through its intracellular segment AICD, thereby promoting the early osteogenic differentiation of MSCs but inhibiting the self-renewal and proliferation abilities of MSCs, resulting in the gradual depletion of MSCs storage and affecting long-term bone formation. In addition, APPswe negatively regulates the migratory ability of MSCs, resulting in impaired MSC homing and affecting bone repair.

## Ethics declaration

All animal experiments were approved by the Institutional Animal Care and Use Committee of Chongqing Medical University (Approval No: IACUC-CQMU-2023-0224).

## Funding

This study was supported by the 10.13039/501100005230Natural Science Foundation of Chongqing, China (No. cstc2021ycjh-bgzxm0137), the Science and Technology Project of Yubei District, Chongqing, China (No. 2021-NS-39), and the Basic Research Incubation Project of the Third Affiliated Hospital of 10.13039/501100004374Chongqing Medical University, Chongqing, China (No. KY20074).

## Author contributions

The study was designed and conceived by Zhou Qiang. The experiments were conducted by Wang Nan, Huakun Huang, Habu Jiwa, Jixing Ye, Zongxin Li, Pei Li, and Runhan Zhao. The manuscript was written by Wang Nan, and the experimental data were prepared and analyzed by Xiaoyu Shen. Huakun Huang performed partial western blot experiments. Habu Jiwa contributed a lot of work in establishing animal model. Runhan Zhao has played an important role in GEO database analysis. Jixing Ye and Pei Li gave important input in the design and guidance of the experiment.

## Data availability

All data generated or analyzed during this study are included in this article. The datasets used and/or analyzed during the current study are available from the corresponding author upon reasonable request.

## Conflict of interests

The authors declared no competing interests.

## Figures and Tables

**figs1:**