# Supplementary material for: The bidirectional effects of APPswe on the osteogenic differentiation of MSCs in bone homeostasis by regulating Notch signaling
Source: Genes Dis. 2024 May 9;12(4):101317. doi: 10.1016/j.gendis.2024.101317 (PMC12052679; doi:10.1016/j.gendis.2024.101317)
Supplement: Multimedia component 1 [file mmc1.doc]

**Supplementary image**


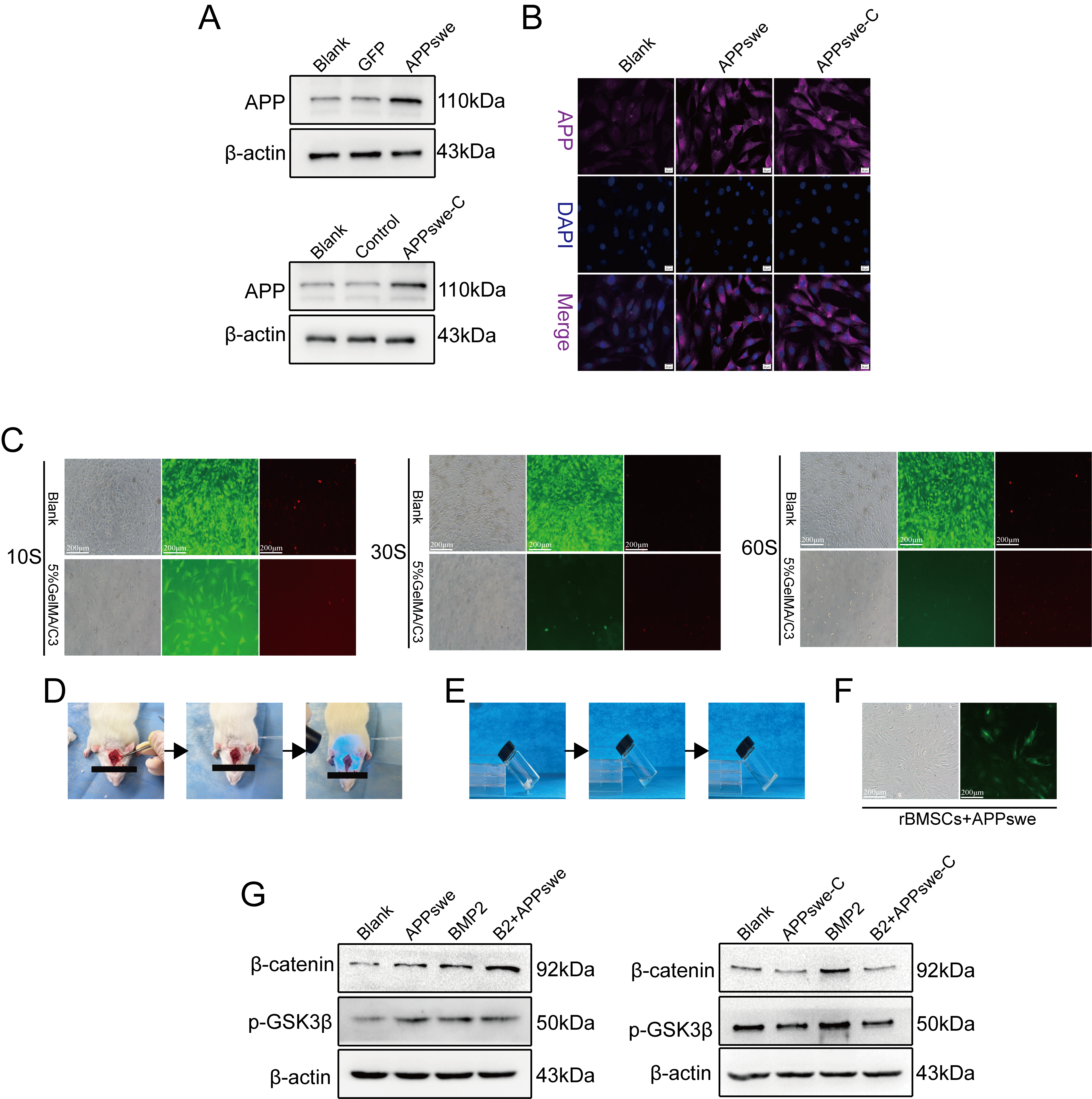


**Figure S1** Supplementary materials for different experimental projects in the study. **(A, B)** The expression levels of APPswe and APPswe-C in MSCs were detected by western blot and cellular immunofluorescence after treatment with APPswe or APPswe-C for 48 h (the picture shows a combination of cells after layer scanning). **(C)** After ultraviolet photocrosslinking for different durations, the survival of cells in the 5% GelMA hydrogel was detected after culturing for 24 h by live/dead staining. **(D)** A critical bone defect model of the rat skull was established, composite hydrogel material was added, and ultraviolet crosslinking was performed. **(E)** GelMA hydrogel material in different forms. **(F)** The transfection efficiency of the APPswe adenovirus into the rBMSCs was detected by fluorescence assay. **(G)** The expression levels of p-GSK-3β (Ser9) and β-catenin were detected by western blotting. MSCs were treated with APPswe or APPswe-C for 24 h and then with BMP2 for 48 h to detect the expression levels of related proteins. APPswe, Swedish mutant amyloid precursor protein; APPswe-C, APPswe without an intracellular segment; MSC, mesenchymal stem cell; BMP2, bone morphogenetic protein 2; GelMA, gelatin methacryloyl.

**
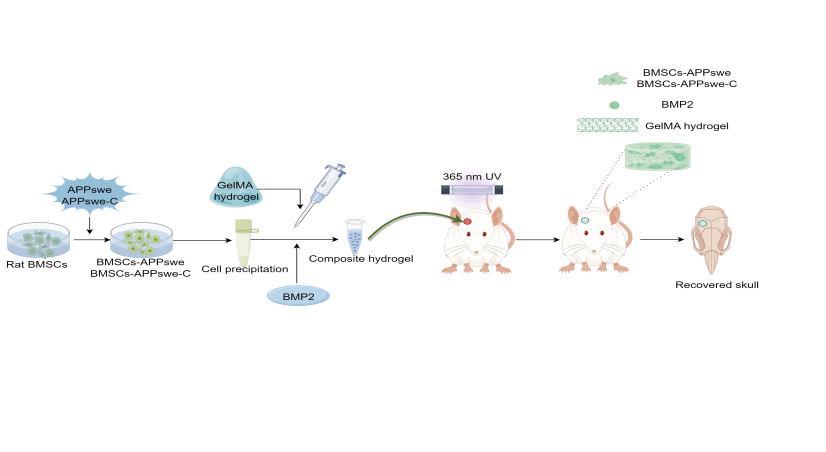
**

**Graphic summary was drawn by Figdraw**
