# Supplementary material for: The bidirectional effects of APPswe on the osteogenic differentiation of MSCs in bone homeostasis by regulating Notch signaling
Source: Genes Dis. 2024 May 9;12(4):101317. doi: 10.1016/j.gendis.2024.101317 (PMC12052679; doi:10.1016/j.gendis.2024.101317)
Supplement: Multimedia component 2 [file mmc2.docx]

1、The data used to support the findings of this study are included within the article.
2、The raw data used to support the findings of this study are available from the corresponding author upon request.
